# Multiple Wire-Mesh Sensors Applied to the Characterization of Two-Phase Flow inside a Cyclonic Flow Distribution System †

**DOI:** 10.3390/s19010193

**Published:** 2019-01-07

**Authors:** César Y. Ofuchi, Henrique K. Eidt, Carolina C. Rodrigues, Eduardo N. dos Santos, Paulo H. D. dos Santos, Marco J. da Silva, Flávio Neves, Paulo Vinicius S. R. Domingos, Rigoberto E. M. Morales

**Affiliations:** 1 Graduate Program in Electrical and Computer Engineering (CPGEI), Federal University of Technology-PR, Curitiba 80230-901, Brazil; e.n.santos@ieee.org (E.N.d.S.); mdasilva@utfpr.edu.br (M.J.d.S.); neves@utfpr.edu.br (F.N.J.); 2 Multiphase Flow Research Center (NUEM), Federal University of Technology-PR, Curitiba 80230-901, Brazil; hkeidt@gmail.com (H.K.E.); carolcimarelli@gmail.com (C.C.R.); psantos@utfpr.edu.br (P.H.D.d.S.); rmorales@utfpr.edu.br (R.E.M.M.); 3 Graduate Program in Mechanical and Materials Engineering (PPGEM), Federal University of Technology-PR, Curitiba 80230-901, Brazil; 4 Petróleo Brasileiro S.A. (Petrobras), Rio de Janeiro 21941-915, Brazil; paulodomingos@petrobras.com.br

**Keywords:** wire-mesh, flow distribution, subsea gas–liquid separation, two-phase flow, cyclonic chamber

## Abstract

Wire-mesh sensors are used to determine the phase fraction of gas–liquid two-phase flow in many industrial applications. In this paper, we report the use of the sensor to study the flow behavior inside an offshore oil and gas industry device for subsea phase separation. The study focused on the behavior of gas–liquid slug flow inside a flow distribution device with four outlets, which is part of the subsea phase separator system. The void fraction profile and the flow symmetry across the outlets were investigated using tomographic wire-mesh sensors and a camera. Results showed an ascendant liquid film in the cyclonic chamber with the gas phase at the center of the pipe generating a symmetrical flow. Dispersed bubbles coalesced into a gas vortex due to the centrifugal force inside the cyclonic chamber. The behavior favored the separation of smaller bubbles from the liquid bulk, which was an important parameter for gas-liquid separator sizing. The void fraction analysis of the outlets showed an even flow distribution with less than 10% difference, which was a satisfactorily result that may contribute to a reduction on the subsea gas–liquid separators size. From the outcomes of this study, detailed information regarding this type of flow distribution system was extracted. Thereby, wire-mesh sensors were successfully applied to investigate a new type of equipment for the offshore oil and gas industry.

## 1. Introduction

Sensing technology for two-phase flow monitoring has evolved from simple visualization techniques and global parameters measurement (such as pressure drop and temperature) to use of tomographic and imaging techniques to discover details of flow behavior in pipes and equipment. The current use of computational power to simulate complex flow and to predict its behavior has also pushed the development of measurement techniques to measure flow parameters with greater detail, i.e., high spatial and temporal resolution [1]. Among tomographic techniques based on a variety of measuring principles—such as gamma-ray, X-ray, impedance, ultrasound and others—a technique known as the wire-mesh sensor has emerged as a very competitive alternative, due to its high spatial and temporal resolution (up to few millimeters and few kilohertz range). It has been applied in a number of pilot plant studies around the world [2,3,4,5,6]. Hence, in this paper, the wire-mesh sensor was applied for the first time to characterize the flow inside reduced-scale subsea equipment from the oil industry.

Offshore deep-water discoveries have driven the interest of the industry in new subsea separation technologies. According to the International Energy Agency, the petroleum withdrawn on platforms represents 30% of all world production. The crude oil of offshore reservoirs is generally mixed with many components, such as liquid water, gas and solids. In this way, devices are used to separate those components, e.g., gravitational separator devices in the offshore platform [7].

In the past years, there is a trend to move the processing unit from the topside to the seabed level, to optimize oil production in deep water environments [8]. Separation of liquid and gas phases at the wellhead level can mitigate the hydrate risk after the separation and increase liquid boosting of submersible centrifugal pumps, or any artificial lift process used, among other advantages. Subsea separation also allows the debottlenecking of topside water treatment (water/hydrocarbon separation and subsea water reinjection) [9]. Gravitational and centrifugal separators are generally used for this purpose due to their high separation efficiency. However, they require large dimensions, which makes the construction, installation and maintenance of this equipment difficult at offshore deep-water applications. 

Numerous alternatives to conventional separators based on gravity and centrifugal flow have been proposed. Separators that use the cyclone concepts as VASPS (Vertical Annular Separation and Pumping System) [10], include the CS (Cyclone Separator) [11,12] and the GLCC© (Gas–Liquid-Cylindrical Cyclone) [13,14]. The concept is based on tangential inlets in vertical pipes to provide the swirling motion to the incoming mixture. The resulting centrifugal force enhances phase separation since the liquid phase flows near the wall and the gas phase flows in the middle of the pipe, which is induced by the difference of density between the phases. Compact GLCC separators are smaller than conventional separators and could reduce the costs of the development of an oilfield. However, reducing the size of separators also reduces the separator robustness to handle fluctuations in the flow rate and composition. Hence, some authors proposed the combination of cyclone devices as pre-separators or as partial separators [15]. Another alternative to solve the problems of conventional cyclonic separators is to reduce its dimensions by distributing the flow to smaller vessels with reduced wall thickness [16]. The distributor ideally will produce symmetrical flow rates across all outlets to optimize the separator dimensions project.

**Figure 1:**
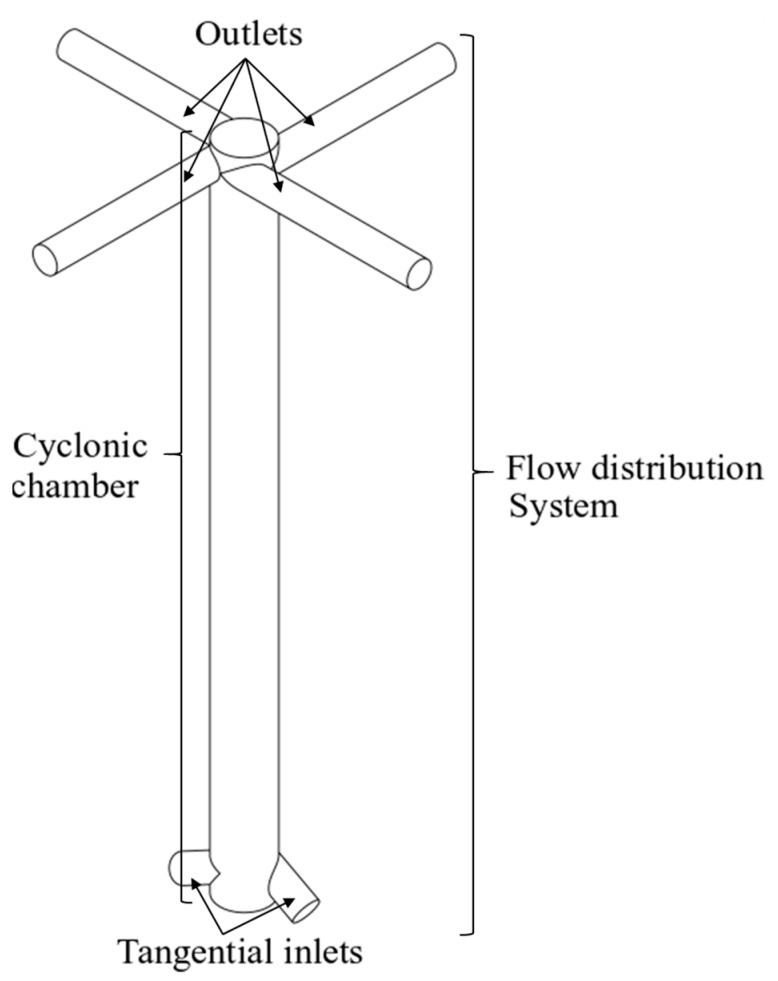
Schematic representation of the flow distribution system.

In this context, a novel design of a flow distributor system, proposed by the authors, is experimentally investigated. Tomographic instrumentation and a camera were applied to evaluate the two-phase flow behavior inside the distributor device presented in Figure 1. A cyclonic chamber, in which the entries are tangentially located at the bottom and outlets at the top, was used as a pre-distribution/separation device. Due to the positioning of the inputs being tangential in the distributor, an ascendant liquid film flow, driven by a centrifugal field, results when the liquid-gas mixture enters the cyclonic chamber. This flow has the characteristic, in which a thin liquid film flows near the wall under the action of centrifugal and gravitational fields until the outlets. The device could be coupled with gas-liquid separators such as the VASPS. The study focused on the slug flow pattern, since it is the worst-case scenario, as the gas and the liquid flow could be unevenly distributed to different separators. Wire-mesh sensors were used to identify the flow pattern and to measure the void fraction at the input, inside the cyclonic chamber and at the four outputs. A camera was also used to capture the flow behavior through the transparent pipeline.

## 2. Experimental Setup

**Figure 2:**
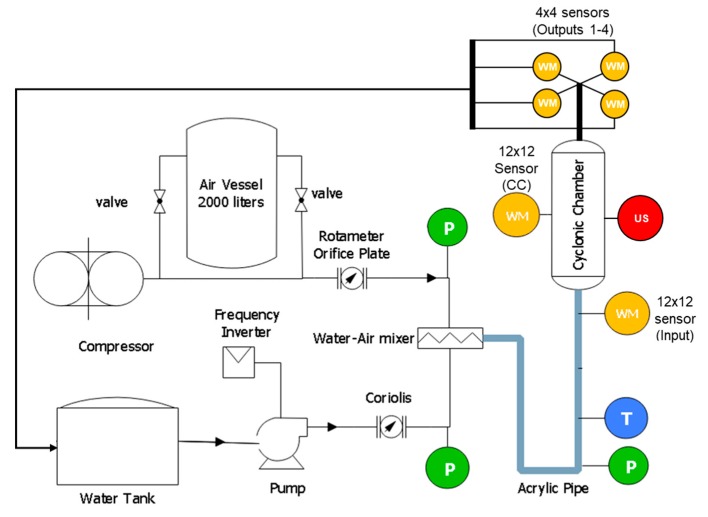
Schematic representation of the measurement plant with the wire-mesh sensors (WMS), ultrasound (US), pressure and temperature sensors (P) and (T).

**Figure 3:**
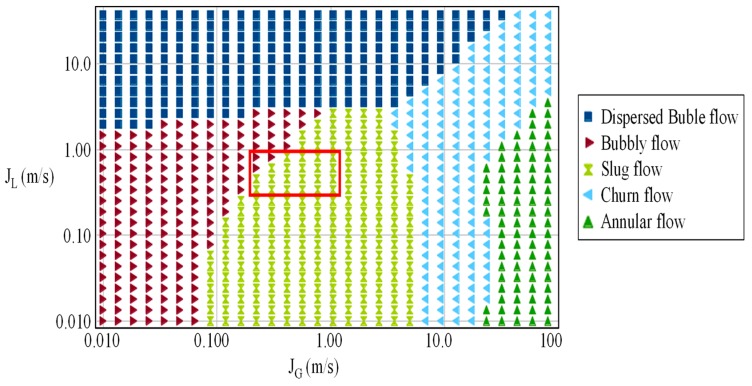
Flow map test setup for slug flow based on [17].

Figure 2 shows a schematic representation of the experimental flow loop used in this work. The water-air mixture flows through an acrylic pipe of 26 mm (1 inch) internal diameter and 7 m in length. Tap water circulates throughout a closed loop using a pump, and air flow is produced by a compressor and stored in a vessel. Air and water flow rates are independently measured by a rotameter and a Coriolis flow meter. The pipe ends in a vertical direction, where the flow is divided into two channels and tangentially enters the cyclonic chamber. The flow is distributed through four outputs with a 13 mm diameter (1/2 inch). The rotameter readings are compensated by the pressure difference at the pipe entrance and the pressure at the measurement point. Temperatures, pressures and flow rates are monitored using industrial sensors connected by Foundation Fieldbus. The flow rate is also controlled by a frequency inverter, by National Instrument System acquisition, using LabVIEW. We wanted to evaluate the slug flow pattern at the input, so we used a flow map (Figure 3), which is a simple method of determining flow regimes based upon a known range of phase velocities. We followed the flow map elaborated by [17] for the setup of gas and liquid superficial velocities (The superficial velocity is normally defined as *Q/A,* where *Q* is the volumetric flow rate (e.g., m^3^/s) of the fluid, and *A* is the cross-sectional area (e.g., m^2^)), as it had the same vertical, 1 inch, water-air two-phase flow as in our experiment. The highlighted rectangle comprises a test setup with superficial gas velocities (*J_G_*) from 0.5 to 1.0 m/s, and superficial liquid velocities (*J_L_*) from 0.5 to 2.0 m/s with a 0.5 m/s step.

To investigate the behavior of the liquid-gas flow inside the distribution system, we analyzed the variation of the void fraction through the system. A wire-mesh sensor (WMS) was used to measure the void fraction [18]. This technique is a reliable flow visualization tool with high spatial and temporal resolution. The sensor consists of transmitter and receiver wires measuring the electrical properties of the flow within its slightly spaced crossing points. The transmitter electrodes are consecutively activated while keeping all other transmitter electrodes at ground potential. The pair of electrodes measured the electrical permittivity of the surrounding flow phase at the crossing point and then converted it to phase fraction, by calibration routines taken before the measurements. More details on the capacitive wire-mesh sensor can be found at [19]. The electronics, commercialized by HZDR Innovation GmbH, was configured with a frame-rate of 1000 Hz. The gas-liquid mixture flows through a 12 × 12 WMS at the system input, then into a 12 × 12 WMS inside the cyclonic chamber, and finally through the four 4 × 4 WMS at the system output, as described in Figure 2. The number of wires is proportional to the pipeline diameter: 1 inch uses 12 × 12 wires and ½ inch uses 4 × 4 wires. A simple camera was used to register the flow image at the same wire-mesh acquisition points as a comparison.

**Figure 4:**
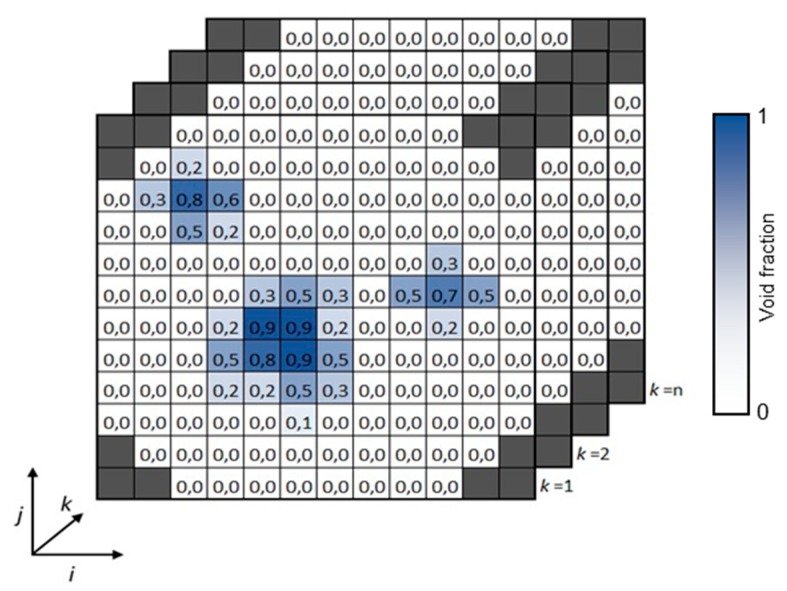
Spatial coordinates of the wire-mesh processing data. Void fraction is represented in the color scale.

The gas fraction α is achieved using the acquired voltages *V* for all crossing points. The values are calibrated using the sensor covered with a high permittivity material *V_H_* (liquid water) and a low permittivity material *V_L_* (air). Equation (1) shows the relation between the variables. Data are stored in a three-dimensional matrix α(*i*,*j*,*k*), where *i* and *j* denote the wire indices and *k* is the temporal sampling point index (Figure 4).

(1) $$\alpha \left(i,j,k\right)=\frac{V\left(i,j,k\right)-V_{L}\left(i,j\right)}{V_{H}\left(i,j\right)-V_{L}\left(i,j\right)}$$

**Figure 5:**
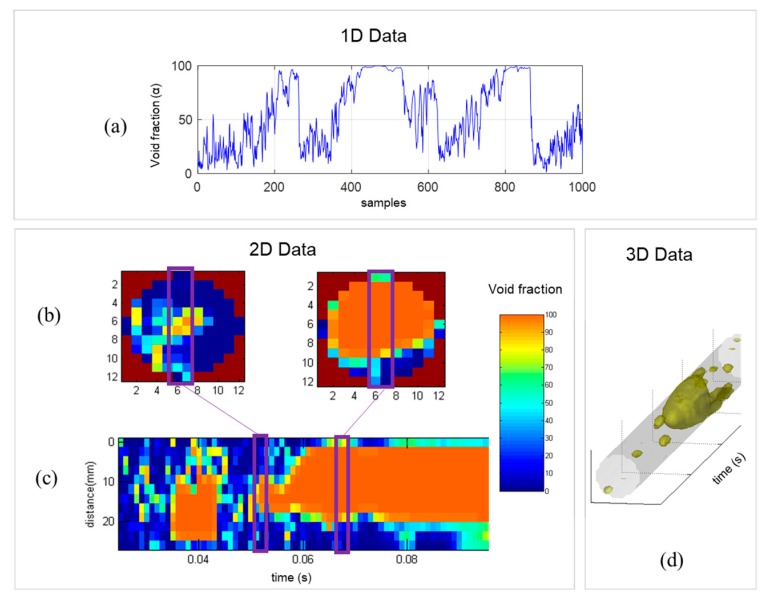
Signal processing of water/gas slug flow acquired with the wire-mesh sensor: (**a**) Averaged void fraction time series, (**b**) cross-sectional and (**c**) axial cut slice images, and (**d**) three-dimensional isosurface plot to view the gas-phase boundaries.

Based on the *α* matrix, one can process the void fraction data to analyze the flow behavior. Figure 5 depicts a different data representation of wire-mesh sensor readings, which are explained by Equations (2)–(4). More details can be found on [19].

We evaluate the overall void fraction behavior of the flow by using the one-dimensional data of the averaged void fraction ($\bar{\alpha }$) time-series, presented in Figure 5a and Equation (2):

(2) $$\bar{\alpha }\left(k\right)=\frac{1}{N_{i}N_{j}}\overset{N_{i}}{\underset{i}{\sum }}\overset{N_{j}}{\underset{j}{\sum }}\alpha \left(i,j,k\right),$$

where *N_i_* and *N_j_* are the total number of samples in each axis. 

To analyze the flow structure in detail we extract a two-dimensional void fraction data of the cross-section ${\bar{\alpha }}_{cross}$, represented by Figure 5b and Equation (3):

(3) $${\bar{\alpha }}_{cross}\left(i,j\right)=\frac{1}{N_{k}}\overset{N_{k}}{\underset{k}{\sum }}\alpha \left(i,j,k\right),$$

where *N_k_* is the total number of time samples. Additional 2D data are obtained by selecting a specific chord, *i_chord_* (which in general, is the central chord), to get an axial cut of the void fraction matrix, as described in Figure 5c and Equation (4):

(4) $$\alpha \left(j,k\right)=\alpha \left(i_{chord},j,k\right).$$

In contrast to making slices, a different way of exploring volumetric data is to view the three-dimensional boundaries. In Figure 5d, we create a polygon using the isosurface data from the *α* matrix and an isovalue (threshold). We use it to view the void fraction structure at the input to confirm the flow pattern. In addition, we can view the flow structure inside the cyclonic chamber, which will be clear in the Results section. 

## 3. Results and Discussion

In the first test, wire-mesh sensors were installed at the input, inside the cyclonic chamber, and at two outlets. We proposed a qualitative analysis of the void fraction flow pattern in all test sections. We used a camera in the same position as the WMS, installed in the cyclonic chamber afterward to compare the flow structure. In the second test, WMS were installed at the input and the four outlets. The flow distribution rate at each outlet was quantitatively analyzed using a set of slug flow patterns at the input. Table 1 shows the wire-mesh setup in each experiment.

### 3.1. Flow Pattern Analysis

**Figure 6:**
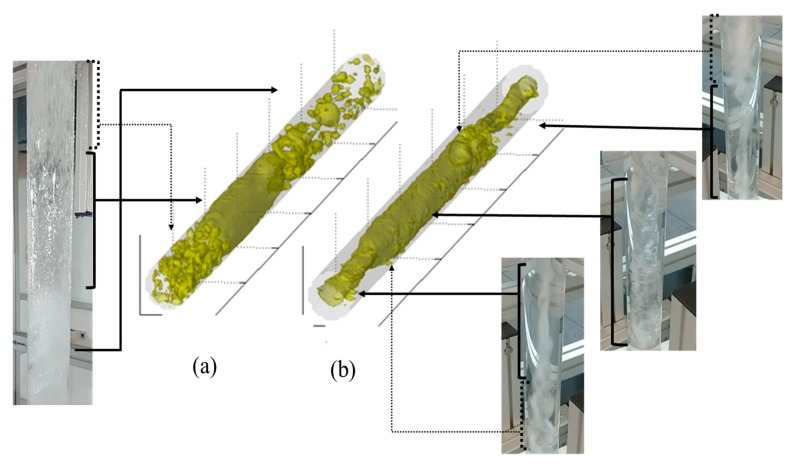
Slug flow images with wire-mesh and camera: (**a**) Input and (**b**) inside the cyclonic chamber.

In this test setup, we evaluated the void fraction of the intermittent flow through the distribution system. The first result was a comparison between the slug flow at the input and inside the cyclonic chamber. Camera and 3D wire-mesh data images are presented in Figure 6.

Figure 6a shows the slug flow pattern at the input for superficial gas velocities (*J_G_*) of 1.5 m/s and liquid velocities (*J_L_*) of 1.0 m/s, which had a dispersed bubbly gas phase followed by a Taylor bubble. The direct tomographic data from the wire-mesh sensor enabled a better view inside the mixture. Figure 6b depicts the flow pattern inside the cyclonic chamber. Centrifugal forces pushed the liquid phase onto the wall of the pipeline with a formation of a gas core in the center. The intermittent gas-liquid flow pattern changed to a rotating flow, where the dispersed bubbles coalesced in a gas vortex.

**Figure 7:**
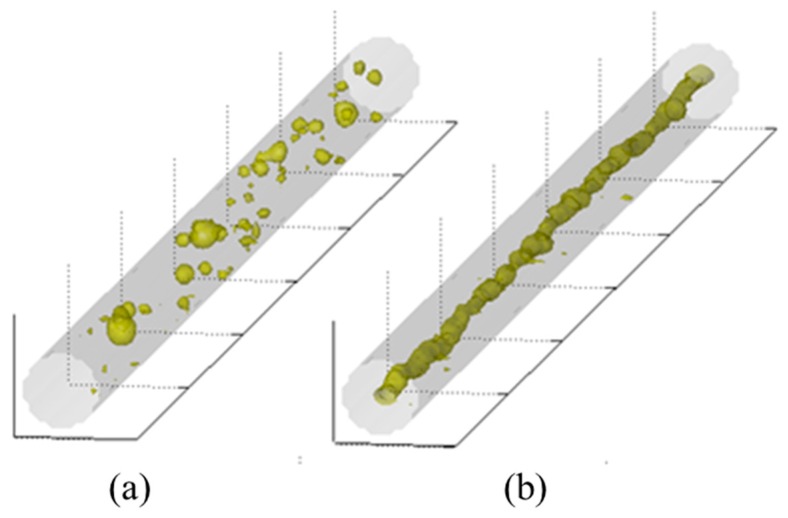
Tomographic wire-mesh data in a dispersed bubbly flow with (*J_G_*) = 0.5 m/s and (*J_L_*) = 1.5 m/s. (**a**) Dispersed bubble at the input and (**b**) gas vortex inside the cyclonic chamber.

The same behavior occurred in another test, with only dispersed bubbles (*J_G_* = 0.5 m/s and *J_L_* = 1.5 m/s), as presented in Figure 7. Wire-mesh data at the input showed the bubbles at the input and the resulting gas vortex inside the cyclonic chamber. This behavior was highly desirable in the gas-liquid separator system context. Most separators are sized to provide enough retention time to allow gas bubbles to form and separate out [20]. Hence, the cyclonic chamber may reduce the separator size, in addition to the flow distribution advantage. 

**Figure 8:**
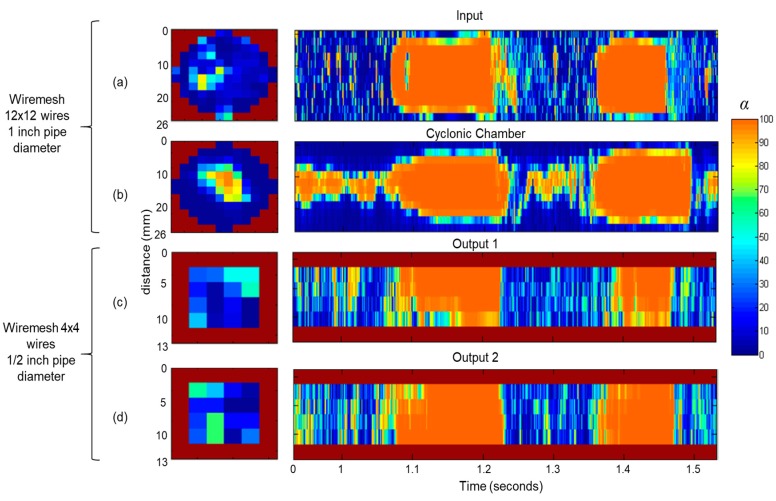
Axial and longitudinal view of void fraction using the WMS in two-phase flow with *J_L_* = 1.0 m/s and *J_G_* = 1.0 m/s. (**a**) Input, (**b**) cyclonic chamber, (**c**) outlet 1 and (**d**) outlet 2.

Flow distribution can be measured based on the instrumented outlets. Figure 8 depicts a detailed axial and longitudinal view of the void fraction measured at the input, inside the cyclonic chamber and at two outlets with the WMS. Original data were synchronized, as the measurement occurs in different test points. 

Figure 8a shows the slug flow pattern at the input. Large gas bubbles were separated by liquid slugs and smaller bubbles were mixed in the liquid slug. The changed flow pattern inside the cyclonic chamber is presented in Figure 8b. Gas and liquid phases maintained the same intermittency as the input. The main difference was the dispersion of the bubbles, inside the liquid slug, which concentrated in the middle of the pipeline due to the centrifugal force. The void fraction at the outlets also followed the slug flow pattern and were equally distributed (Figure 8c,d). Besides the symmetry in flow distribution, the synchronized comparison also showed that the intermittent behavior propagated from the input to the outlets. We also observed the mean void fraction behavior in the input, inside the cyclonic chamber and at the two outlets as the liquid and gas superficial velocities increased. 

**Figure 9:**
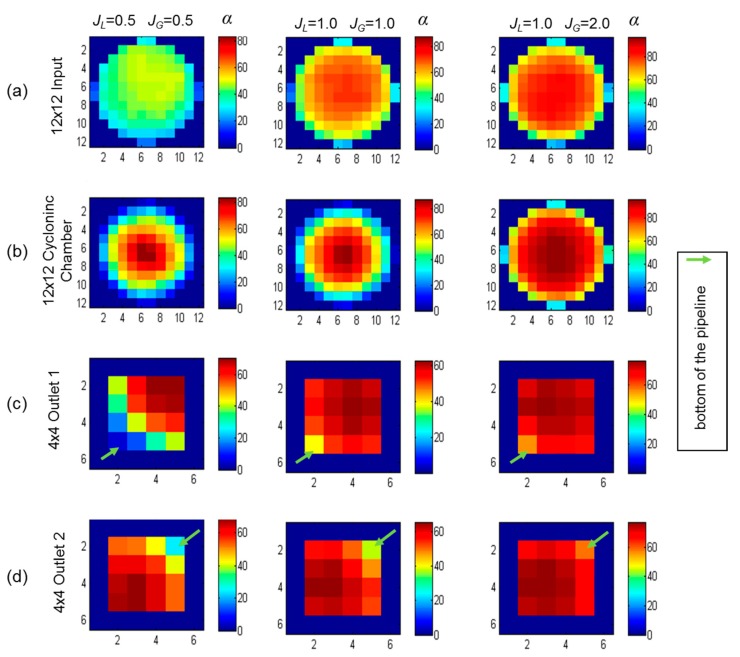
Axial view of the mean void fraction by WMS for three sets of gas and liquid superficial velocities: (**a**) Input using 12 × 12 WMS, (**b**) inside the cyclonic chamber using 12 × 12 WMS, (**c**) outlet 1 using 4 × 4 WMS, and (**d**) outlet 2 using 4 × 4 WMS. The bottom of the pipeline is indicated in the outlets.

Figure 9 shows the mean void fraction measured by the WMS for three sets of superficial velocities: *J_L_* = 0.5/*J_G_* = 0.5, *J_L_* = 1.0/*J_G_* = 1.0 and *J_L_* = 1.0/*J_G_* = 2.0. Figure 9a depicts the void fraction at the input and Figure 9b inside the cyclonic chamber using 12 × 12 WMS. The mean gas phase was dispersed at the input and more concentrated in the center of the pipeline, inside the cyclonic chamber with a liquid film around. The effect was better observed in lower gas velocities. In higher gas velocities, the gas phase almost occupied the entire pipeline. With *J_L_* = 1.0 and *J_G_* = 2.0, the mean void fraction profile at the input was almost the same as inside the cyclonic chamber. Figure 9c,d presents the void fraction in the outlets. As they were horizontally oriented, the liquid phase stayed at the bottom of the pipeline. As already observed, both outlets had the same void fraction profile.

### 3.2. Flow Distribution Analysis

**Figure 10:**
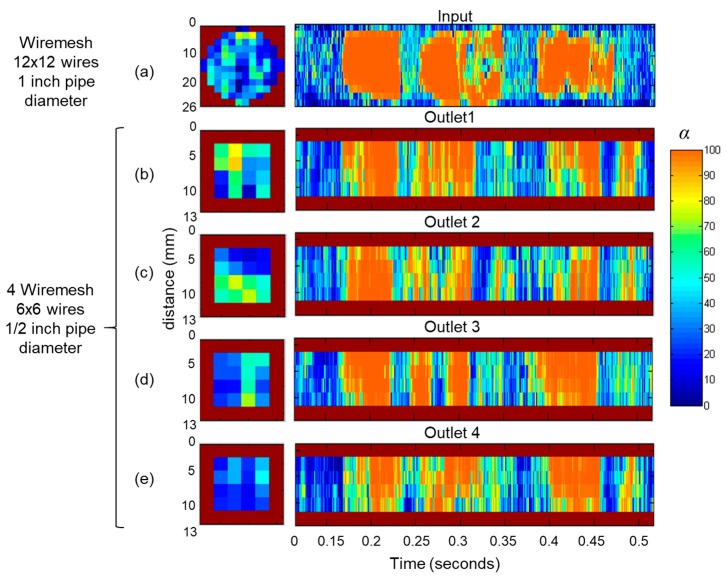
Axial and transversal view of the void fraction, using the wire-mesh sensor in two-phase flow with *J_L_* = 1.0 m/s and *J_G_* = 1.0 m/s. (**a**) Input and (**b**–**e**) outlets 1–4.

In this test setup, the WMS was installed at the input and at the four outlets to verify the flow distribution, according to the input slug flow pattern (Figure 10). Original time series was synchronized, as the measurement occurs in different test points. A slug flow of *J_L_* = 1.0 and *J_G_* = 1.0 was observed at the input (Figure 10a) and propagated through the outlets (Figure 10b–e) with the same liquid-gas profile. The flow distribution was visually symmetrical in the outlets. 

**Figure 11:**
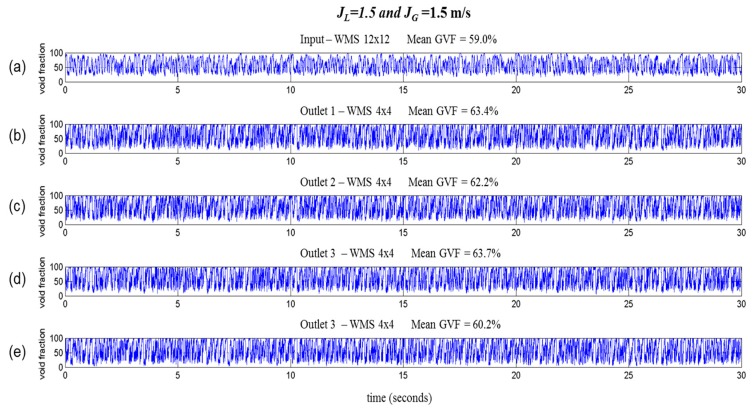
Void fraction time series (30 s. *J_L_* = 1.5 m/s and *J_G_* = 1.5 m/s): (**a**) Input and (**b**–**e**) outlets 1 to 4.

A longer void fraction time-series of 30 s, using superficial gas and liquid velocities of 1.5 m/s is presented in Figure 11. In this setup, numerous gas pockets followed by liquid bulks were observed, which enabled a reasonable quantitative analysis of the flow distribution. The void fraction at the input (59%) agreed with the mean void fraction at the outlets (62.40%). The distributed void fraction between the outlets was almost the same, with a worst-case difference of approximately 3.5% between outlet 3 (63.7%) and outlet 4 (60.2%). 

**Figure 12:**
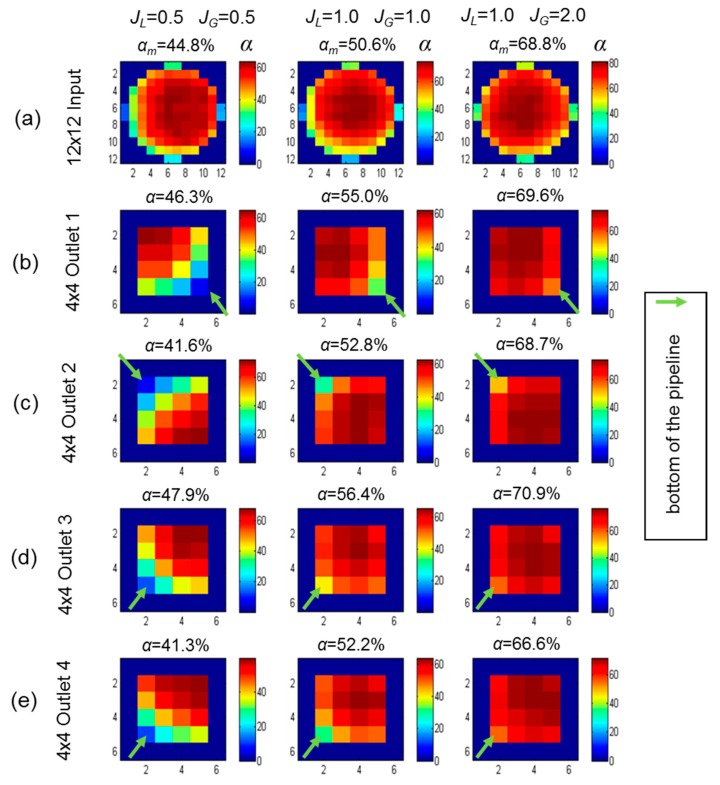
Mean axial void fraction (α_m_) measured in 30 s by WMS in three set of superficial velocities: *J_L_* = 0.5 m/s and *J_G_* = 0.5 m/s; *J_L_* = 1.0 m/s and *J_G_* = 1.0 m/s; and *J_L_* = 1.0 m/s and *J_G_* = 2.0 m/s. (**a**) Input and (**b**–**e**) outlets 1 to 4. WMS data at the outlets are rotated due to a different installation.

We also compared the mean void fraction profile in three different sets of velocities: *J_L_* = 0.5 m/s and *J_G_* = 0.5 m/s; *J_L_* = 1.0 m/s and *J_G_* = 1.0 m/s; *J_L_* = 1.0 m/s and *J_G_* = 2.0 m/s (Figure 12). The mean void fraction at the input increased from 44.8% to 68.8%, as the superficial gas velocity increased from 0.5 m/s to 2 m/s. An important result was the similarity between the void fraction profiles of the four outlets, even with different superficial velocities. 

The complete result is summarized in Table 2, where eleven different sets of superficial gas and liquid velocities are evaluated. These results show a void fraction difference between the outlets to a worst-case difference of 8.2%. Using this flow distribution system, four smaller separators with ¼ of the original size may be used with only an additional tolerance of 10% due to the uneven flow distribution. The difference between the outlet’s decay at higher flow rates (*J_L_* ≥ 1.0 m/s and *J_G_* ≥ 1.0 m/s), allowing a tolerance of around 5%. 

Despite using the same sensors in the outlets, we needed to consider the effects of the 4 × 4 WMS uncertainties. According to [21], WMS can measure the void fraction within 11%—relative to other methods—but the accuracy can be better, depending on the flow pattern. There was also a spatial resolution issue, as WMS pitch effects caused differences in the order of 1 to 4% for bubbly flows, but up to 20% peak at a low void fraction. In future work, we may use a higher resolution WMS and also evaluate the liquid flow rate after the separation process to enhance this study.

## 4. Conclusions

This work presents an experimental analysis of a flow distribution system for gas-liquid separation, using tomographic wire-mesh sensors. The study focused on a qualitative analysis of the slug flow pattern at the input, its behavior through the cyclonic chamber, and the flow distribution symmetry across the outlets. Use of wire-mesh sensors and a camera enabled the visualization of complete flow inside the cyclonic chamber. Results showed an ascendant liquid film in the cyclonic chamber with the gas phase at the center of the pipe generating a symmetrical flow. Dispersed bubbles coalesced into a gas vortex, due to the centrifugal force inside the cyclonic chamber. The behavior favored the separation of smaller bubbles from the liquid bulk, which was an important parameter for gas-liquid separator sizing. The void fraction analysis of the outlets showed an even flow distribution with less than 10% difference. At higher flow rates, the distribution rate difference decayed to 5%. A part of the observed difference could be due to the 4 × 4 WMS uncertainties. In future work, we may use a higher resolution WMS and also evaluate the liquid flow rate after the separation process. In summary, these results contribute to a new flow distribution system design, which is suitable for subsea gas-liquid separation problems. Hence, wire-mesh sensors were successfully applied to investigate a case study of the offshore oil and gas industry.

## Figures and Tables

**Table 1:** Arrangement of wire-mesh sensors in each test.

Test	Input	Cyclonic Chamber	Outlet
Flow pattern analysis	12 × 12	12 × 12	Two 4 × 4
Flow distribution analysis	12 × 12	--	Four 4 × 4

**Table 2:** Summary of mean void fraction results.

*J_L_* (m/s)	*J_G_* (m/s)	Void Fraction (%)							
		Input	Outlet 1	Outlet 2	Outlet 3	Outlet 4	Mean	Worst Case Difference	
		12 × 12	4 × 4	4 × 4	4 × 4	4 × 4	Outlets	Value	%
0.5	0.5	44.84	46.30	41.63	47.91	41.34	44.29	3.61	8.2
0.5	1.0	60.18	64.22	60.23	65.66	58.17	62.07	3.90	6.3
1.0	0.5	33.08	40.68	38.67	42.01	37.78	39.79	2.22	5.6
1.0	1.0	50.59	55.04	52.82	56.47	52.29	54.15	2.32	4.3
1.0	1.5	62.03	63.63	62.27	65.20	60.54	62.91	2.38	3.8
1.0	2.0	68.83	69.62	68.70	70.93	66.67	68.98	2.31	3.3
1.5	0.5	29.93	40.63	38.41	41.44	37.88	39.59	1.85	4.7
1.5	1.0	47.69	53.48	52.59	54.96	50.44	52.87	2.42	4.6
1.5	1.5	59.00	63.41	62.19	63.79	60.21	62.40	3.20	5.1
2.0	0.5	30.80	44.14	44.32	46.14	41.93	44.13	2.20	5.0
2.0	1.0	45.49	56.99	55.71	57.80	54.73	56.31	1.58	2.8
